# Genetic strategies for enhancing litter size and birth weight uniformity in piglets

**DOI:** 10.3389/fvets.2025.1512701

**Published:** 2025-03-24

**Authors:** Wuttigrai Boonkum, Suwanee Permthongchoochai, Vibuntita Chankitisakul, Monchai Duangjinda

**Affiliations:** ^1^Department of Animal Science, Faculty of Agriculture, Khon Kaen University, Khon Kaen, Thailand; ^2^Network Center of Animal Breeding and Omics Research, Khon Kaen University, Khon Kaen, Thailand

**Keywords:** genetic correlation, heritability, piglet uniformity, number born alive, swine

## Abstract

This study aimed to estimate the genetic parameters and develop selection indices for litter size and birth weight uniformity in piglets. These traits are crucial for improving productivity and profitability of swine production. Data were collected from 9,969 litters of 4,465 sows and 106,463 piglets of various breeds from a farm in Thailand. The analyzed traits included the total number born (TNB), number born alive (NBA), litter birth weight (LBW), mean birth weight, and individual birth weight. The assessed piglet uniformity traits included the difference between the maximum and minimum values (range), interquartile range of birth weight (IQRBW), variance in birth weight (VBW), standard deviation of birth weight (SDBW), and coefficient of variation of birth weight (CVBW). Variance components and genetic parameters were estimated using a multiple-trait animal model and the average information-restricted maximum likelihood method. The appropriate selection index (I) was determined based on heritability, genetic correlations between traits, and the economic significance of the traits. The results revealed that litter size traits (TNB and NBA) and piglet birth weight uniformity traits (Range, IQRBW, VBW, SDBW, and CVBW) exhibited low heritability (*p* < 0.1), suggesting that environmental factors have a substantial influence. In contrast, birth weight traits showed moderate heritability (approximately 0.2). Negative genetic correlations between litter size and birth weight traits were observed, indicating that increasing litter size might reduce piglet birth weight and uniformity, potentially affecting survival rate. A selection index combining NBA, LBW, and CVBW was constructed to optimize the selection process for productivity and uniformity. In conclusion, genetic improvement programs should prioritize litter size and birth weight uniformity to enhance productivity and uniformity on commercial pig farms. These findings can assist breeders in developing more effective selection strategies, ultimately resulting in larger, more uniform litters and improved overall farm efficiency.

## 1 Introduction

The primary objective of selecting maternal lines in pigs is to improve sow productivity. Litter size refers to the total number of offspring born per litter, commonly measured as total number born (TNB) or number born alive (NBA). It is a key reproductive trait in livestock production, directly influencing the efficiency and profitability of breeding programs. Larger litters increase the potential for higher weaning weights and overall production output, making it an economically significant trait for livestock producers (1, 2). Moreover, the uniformity of piglets within a litter is equally important, as it affects their health, growth performance, and marketability (3–5). In general, uniformity in birth weight refers to the consistency of individual piglet weights within a litter. Litter size and uniformity in pigs can vary across different breeds. For instance, Zhao et al. (6) reported that the mean total number born and individual piglet birth weight were 14.40 and 1.31 kg in Landrace, and 15.27 and 1.27 kg in Yorkshire. Sell-Kubiak (7) found that the average total number born and individual piglet birth weight was 13.65 and 1.25 kg in the Large White pig population in the Netherlands. Zaalberg et al. (8) presented data on purebred Landrace and crossbred Yorkshire × Landrace sows and piglets in Denmark, revealing average values of 15.16 for total number born and 1.57 kg for individual piglet birth weight. Furthermore, Bunz and Harper (9) compared litters with fewer than 10 piglets and more than 17 piglets in a pig farm in Australia, noting a reduction of 0.31 kg per piglet and a 4.8% increase in birth weight variation.

Current research on improving sow reproductive performance focuses on increasing litter size while maintaining uniform piglet birth weight. To assess birth weight variation within a litter, studies commonly use measurement methods such as standard deviation (SDBW), variance (VBW), coefficient of variation (CVBW), or interquartile range (IQRBW) (10–12). Uniformity is crucial for balancing the competition among littermates, improving survival rates, and ensuring more consistent growth performance (13). Greater uniformity enhances management efficiency by simplifying nutritional and healthcare interventions, ultimately contributing to better animal welfare and increased production efficiency. Both traits—litter size and uniformity—are critical for optimizing reproductive success and production outcomes in livestock systems. Together, they represent a balance between maximizing productivity (through larger litter sizes) and ensuring sustainability (through improved uniformity).

However, selecting and genetically improving sows for larger litter sizes has been shown to result in piglets with lower birth weights and increased size variation (14, 15). This is because an increase in litter size obtained with genetic selection has been associated to lower individual birth weights as well as greater variation in birth weight within litter. In terms of genetic parameter estimation, heritability estimates and genetic correlations, play a crucial role in understanding the genetic architecture of litter size and uniformity in birth weight traits. Heritability estimates for litter size traits, such as the total number born (TNB) and the number born alive (NBA), are typically moderate to low, ranging from 0.10 to 0.20 in most pig populations (16–18). This indicates that genetic improvement in litter size through selection is achievable but may require several generations to achieve substantial gains. In contrast, heritability estimates for uniformity traits, such as variance in birth weight (VBW), standard deviation of birth weight (SDBW), and the coefficient of variation of birth weight (CVBW), tend to be lower, often below 0.10 (6). These low estimates suggest that uniformity traits are strongly influenced by environmental factors, making genetic improvement more challenging. However, their inclusion in breeding programs can still contribute to reducing variability and improving overall litter performance. Genetic correlations between litter size and uniformity traits are generally unfavorable but not prohibitive. For example, an increase in litter size is often associated with greater variability in birth weight due to resource competition among piglets. Estimates of genetic correlations between TNB or NBA and uniformity traits (e.g., CVBW or SDBW) range from moderate to high and are usually positive (10). This indicates that selection for larger litters may increase variability in piglet birth weight, potentially affecting piglet survival and growth. To address this, multiple-trait selection approaches that consider both litter size and uniformity traits are essential (7, 18). By leveraging these genetic correlations and balancing selection objectives, breeding programs can achieve a more sustainable improvement in both traits, ensuring enhanced productivity and welfare in swine production systems.

At the same time, the selection index is a powerful tool used to optimize genetic improvement by integrating economic values and genetic parameters for multiple traits (19). In the context of litter size and birth weight uniformity, the selection index allows breeders to balance the economic importance of these traits while accounting for their genetic correlations. By assigning relative economic weights to litter size and uniformity traits, the index ensures that selection decisions maximize overall economic returns. By using advanced genetic analysis techniques, researchers can identify specific genes and alleles associated with these traits, facilitating the development of targeted breeding strategies. Previous studies have shown that genetic approaches are the most effective for achieving sustainable results. Kapell et al. (20) examined genetic parameters related to piglet survival, litter size, and IBW in several sire and dam lines of purebred pigs in China and Brazil and concluded that selecting for all three traits provides better outcomes. Banville et al. (21) studied genetic parameters for litter size, piglet growth, and sow early growth and body composition in the Chinese–European Tai Zumu line, and concluded that estimating genetic correlations between litter size and piglet growth traits is essential and should be incorporated into pig breeding programs. Metodiev et al. (22) conducted a genome-wide association study on litter size and weight traits in purebred Yorkshire sows, identifying different genomic regions and potential candidate genes associated with these traits across first and second parity.

Therefore, genetic improvement practices, when combined with management interventions, offer a more effective and sustainable approach to optimizing litter size, uniformity, survivability, and early growth in pigs. From these reasons, we aimed to estimate the genetic parameters of litter size and birth weight uniformity using a large dataset. We hope that the results of this study will provide precise guidelines for future breeding programs.

## 2 Materials and methods

### 2.1 Animal management and data collection

Data were collected from a commercial farm in central Thailand between 2012 and 2014. A total of 9,969 litters from 4,465 sows (Large White, Landrace, Large White × Landrace crossbred, and synthetic crossbred) and 106,463 piglets were analyzed, including records of TNB, NBA, litter birth weight (LBW), mean birth weight (MBW), and IBW. The farm’s mating system is based on artificial insemination using chilled semen. Each pig was vaccinated against foot-and-mouth disease and swine fever following the vaccination program established by the Thai Department of Livestock Development. The pigs were housed in a closed system equipped with an evaporative cooling system, providing a controlled environment with an average of 12 h of natural light daily to support their health and productivity. For gestating sows, the air temperature was maintained between 18–22°C, while lactating sows thrived in a range of 16–20°C. The temperature for newborn piglets was kept between 32–35°C during the initial days, gradually decreasing to 26–28°C by the third week. Relative humidity was maintained between 50–70%. In this study, the birth period for sows ranged from 3 to 8 h, with piglets typically being delivered within this timeframe. The sow feeding program included three distinct formulas, each with varying ingredients. However, all formulas were standardized to provide 14% crude protein and 3,160 kcal of metabolizable energy (ME) per kilogram. The dietary composition of the sows is presented in Table 1. Sows had unrestricted access to fresh water throughout the study. Piglet uniformity traits within each litter were assessed using several measures, including the difference between the maximum and minimum values (range), interquartile range of birth weight (IQRBW), variance in birth weight (VBW), standard deviation of birth weight (SDBW), and coefficient of variation of birth weight (CVBW). The data used in this study consisted of a pedigree file and a data file containing the following details: sow ID, sire of sow ID, sow birth date, mating boar ID, farrowing date, parity, breed of sow, breed of piglet, sex ratio of piglets (male-to-female), sow body condition score, and sow feeding program. The summary statistics for the studied traits are shown in Table 1. The equations for the traits related to piglet uniformity are as follows:


Range=Maximum–Minimum

IQRBW=Quartile3−Quartile1

$$\mathrm{VBW}=\frac{\sum {\left(X_{i}-\bar{X}\right)}^{2}}{n-1}$$

$$\mathrm{SDBW}=\sqrt{\frac{\sum {\left(X_{i}-\bar{X}\right)}^{2}}{n-1}}$$

$$\mathrm{CVBW}=\frac{\mathrm{SD}}{\bar{X}}\times 100$$

**Table 1:** The dietary composition of the sows in this study.

Ingredient	Sow dietary composition		
	1st dietary composition (%)	2nd dietary composition (%)	3rd dietary composition (%)
Moisture	11.50	12.36	10.85
Ash	3.90	7.33	7.80
Starch	52.00	51.73	46.55
Total lysine	0.72	0.54	0.82
Calcium	1.2	0.89	1.13
Phosphorus	0.68	0.87	0.91
Crude fat	5.3	5.4	4.35
Crude fiber	3.7	7.06	5.10
Crude protein	14	14	14
ME kcal/kg	3,160	3,160	3,160

where maximum and minimum are the largest and smallest values in the dataset, quartile 3 and quartile 1 are the value below which 75% (the third quartile) and value below which 25% of the data set (the first quartile), $X_{i}$ is the birth weight of piglet i−th in the dataset, $\bar{X}$ is the average birth weight of all piglets in the dataset, n is the total number of piglets, SD is the standard deviation value of birth weight of piglet.

### 2.2 Genetic analysis

Before analyzing genetic parameters, the normality of the data was assessed using the Shapiro–Wilk test on the unadjusted phenotypic values, and Levene’s test was used to assess the homogeneity of variance across different groups defined by fixed effects, including sow breed, piglet breed, parity, mating boar, farrowing month-year, sex ratio of piglets, sow body condition score, and sow feeding program. Outliers were identified and removed using Z-scores before analysis. Variance components and genetic parameters (heritability, genetic correlations, and phenotypic correlations) for all 10 traits were estimated simultaneously using a multiple-trait animal model with the averaged restricted information maximum likelihood method, implemented in the BLUPF90+ program (23). To ensure robustness, we carefully selected appropriate initial prior variance values. The program first estimated the initial variance using the EM-REML method for approximately 100 iterations to obtain reliable starting prior values, which were then used for further analysis with the AI-REML method. Additionally, we closely monitored convergence diagnostics to ensure accurate and reliable parameter estimation. Table 2 shows the genetic models used in the analysis. The multiple-trait animal model used was represented by the following equations:


y=Xβ+Zd+Ss+Wp+e

**Table 2:** Description of models used for litter size, piglet birth weight, and uniformity of piglet birth weight traits.

Traits	Litter size traits		Piglet birth weight traits			Uniformity of piglet birth weight traits				
	TNB	NBA	LBW	MBW	IBW	Range	IQRBW	VBW	SDBW	CVBW
Fixed effect										
Breed of piglets	×	×	×	×	×	×	×	×	×	×
Sex ratio between male and female piglets	●	●	●	●	×	×	×	×	×	×
Body condition score of sows	×	×	×	×	×	×	×	×	×	×
Breed of sows	×	×	×	×	×	×	×	×	×	×
Parity of sows	×	×	×	×	×	×	×	×	×	×
Month-year of farrowing	×	×	×	×	×	×	×	×	×	×
Sow feeding program	×	×	×	×	×	×	×	×	×	×
Random effect										
Direct additive	×	×	×	×	×	×	×	×	×	×
Mate-sire	×	×	●	●	●	●	●	●	●	●
Maternal	●	●	×	×	×	×	×	×	×	×
Permanent environment	×	×	×	×	●	×	×	×	×	×
Residual	×	×	×	×	×	×	×	×	×	×

× = factor used in the model; ● = factors not used in the model because no statistical differences were found at *p* > 0.05.

for litter size traits (TNB and NBA).


y=Xβ+Zd+Mm+Wp+e

for piglet birth weight and uniformity of piglet birth weight traits (LBW, MBW, IBW, Range, IQRBW, VBW, SDBW, CVBW).

where y is the vector corresponding to the observation values of TNB, NBA, LBW, MBW, IBW, Range, IQRBW, VBW, SDBW, and CVBW traits, β is the vector of fixed effects, which includes the piglet breed class, parity class, sow body condition score class, and the sex ratio between male and female piglets class. The contemporary group effects include the sow breed class, month-year of farrowing class, and sow feeding program class, d is the vector of random direct additive genetic effects, assumed to be $d\sim N\left(0,\;A\sigma_{d}^{2}\right),$ where A is an additive genetic relationship matrix using pedigree information and $\sigma_{d}^{2}$ is the direct additive genetic variance, s is the vector of random mate-sire effects, assumed to be $s\sim N\left(0,\;A\sigma_{s}^{2}\right),$ where $\sigma_{s}^{2}$ is the mate-sire genetic variance, m is the vector of random maternal effects, assumed to be $m\sim N\left(0,\;A\sigma_{m}^{2}\right),$ where $\sigma_{m}^{2}$ is the maternal genetic variance, p is the vector of random permanent environmental effects, assumed to be $p\sim N\left(0,\;I\sigma_{p}^{2}\right),$ where I is an identity matrix and $\sigma_{p}^{2}$ is the permanent environmental variance, e is the vector of random residual effects assumed to be $e\sim N\left(0,\;I\sigma_{e}^{2}\right),$ where $\sigma_{e}^{2}$ is the residual variance, X,Z,S,M,W are incidence matrices related to the vectors β,d,s,m,p, respectively.

The variance–covariance matrix for all models were:


$$V\left[\begin{array}{c} d\\ \begin{array}{c} m\\ s\\ \begin{array}{c} p\\ e \end{array} \end{array} \end{array}\right]=\left[\begin{array}{ccccc} A⊗G_{d} & A⊗G_{d,m} & 0 & 0 & 0\\ A⊗G_{d,m} & A⊗G_{m} & 0 & 0 & 0\\ 0 & 0 & A⊗G_{s} & 0 & 0\\ 0 & 0 & 0 & I⊗P & 0\\ 0 & 0 & 0 & 0 & I⊗R \end{array}\right]$$

where A and I are the additive genetic relationship matrix and identity matrix, respectively. $G_{d}$, $G_{s}$, $G_{m},G_{d,m}$, P and R are the matrices of variances for direct, mate-sire, maternal, covariances between direct and maternal, permanent environment, and residual effects, respectively.

### 2.3 Constructing the selection index

Selection index (I) was calculated using the estimated breeding values (EBV) of litter size (TNB, NBA), piglet birth weight (LBW, MBW, IBW), and uniformity of piglet birth weight (Range, IQRBW, VBW, SDBW, CVBW) traits. The index weights (b) for the selection index were derived using the phenotypic variance–covariance matrix (P), the genetic variance–covariance matrix (G), and the marginal economic values (MEV) of the traits according to the following formula: $b=P^{-1}\mathrm{Gv}$. The MEV of a trait refers to the change in economic profit resulting from a one-unit increase in that trait, assuming all other traits remain constant. MEV is calculated using the following formula: ${\mathrm{MEV}}_{i}=\frac{\Delta \mathrm{Profit}}{\Delta {\mathrm{Trait}}_{i}}.$After calculating the MEVs, they were adjusted using genetic parameters to ensure proper weighting in the selection index. This adjustment was performed using the following formula: $b_{i}=\frac{{\mathrm{MEV}}_{i}\times h_{i}^{2}}{\sqrt{\sigma_{\mathrm{ai}}^{2}}}$ where $h_{i}^{2}$ = heritability of the trait and $\sigma_{\mathrm{ai}}^{2}$ = additive genetic variance of the trait.

The selection index can be written as:


$$I=\left(b_{1}\times {\mathrm{EBV}}_{\mathrm{trait}1}\right)+\left(b_{2}\times {\mathrm{EBV}}_{\mathrm{trait}2}\right)+\left(b_{3}\times {\mathrm{EBV}}_{\mathrm{trait}3}\right)$$

where I is the selection index; b1, b2, b3 are index weights for litter size, piglet birth weight, and uniformity of piglet birth weight, respectively; and ${\mathrm{EBV}}_{\mathrm{trait}1},{\mathrm{EBV}}_{\mathrm{trait}2},\mathrm{and}\;{\mathrm{EBV}}_{\mathrm{trait}3}$ are estimated breeding values for the traits.

## 3 Results

### 3.1 Descriptive statistics

Descriptive statistics for litter size, piglet birth weight, and uniformity of piglet birth weight in the large dataset are shown in Table 3. For the entire dataset, the mean for TNB, NBA, LBW, MBW, IBW, Range, IQRBW, VBW, SDBW, and CVBW were 11.44, 10.68, 15.96, 1.52, 1.49, 0.79, 0.30, 0.58, 0.16, and 11.03, respectively. The data did not follow a normal distribution, as indicated by the Shapiro–Wilk test (*p* = 0.023), which led to the rejection of the null hypothesis of normality—in terms of skewness and kurtosis, TNB, NBA, LBW, and IBW exhibited left-skewed distributions (negative skewness). In contrast, MBW, Range, IQRBW, VBW, SDBW, and CVBW exhibited right-skewed distributions (positive skewness). Additionally, most traits demonstrated high variability, with a coefficient of variation (CV) exceeding 20%, except for MBW, which had a CV of 16.23%. The wide range of values (maximum-minimum) observed for each trait suggested considerable variability in the weights of individual piglets within the litter.

**Table 3:** Data structure and descriptive statistics for analysis.

Category	Breeds of piglet (sire × dam)							The entire dataset					
	Landrace × Large white	Large white × Landrace	Landrace × 2 crossbred	Large white × 2 crossbred	Synthetic × Landrace	Synthetic × Large white	Synthetic × 2 crossbred	Numbers and overall mean	SD	CV	Min – Max	Skewness	Kurtosis
Number of animals													
Sows (*n*)	613	950	1,259	880	1,093	698	866	4,465	-	-	-	-	-
Boars (*n*)	38	37	43	34	36	38	40	141	-	-	-	-	-
Litters (*n*)	1,107	1,634	1,899	1,120	1,863	1,143	1,203	9,969	-	-	-	-	-
Total born (*n*)	12,715	17,517	21,827	12,762	20,936	13,667	14,637	114,061	-	-	-	-	-
Born alive (*n*)	11,968	16,479	20,224	11,890	19,486	12,742	13,674	106,463	-	-	-	-	-
Litter size traits (mean)													
TNB (*n*)	11.49	10.72	11.49	11.39	11.24	11.96	12.17	11.44	2.70	23.63	4–22	−0.28	0.24
NBA (*n*)	10.81	10.09	10.65	10.62	10.46	11.15	11.37	10.68	2.67	25.02	4–22	−0.29	0.00
Piglet birth weight traits (mean)													
LBW (kg)	15.12	15.25	15.07	15.43	16.88	16.83	17.36	15.96	3.99	25.02	2.5–31.9	−0.11	0.05
MBW (kg)	1.42	1.53	1.43	1.47	1.64	1.54	1.55	1.52	0.25	16.23	0.5–2.5	0.42	0.47
IBW (kg)	1.40	1.51	1.42	1.45	1.61	1.51	1.53	1.49	0.35	23.22	0.3–3.0	−0.02	0.10
Uniformity of piglet birth weight (mean)													
Range	0.75	0.76	0.77	0.78	0.84	0.82	0.83	0.79	0.30	37.48	0.1–2.3	0.34	−0.04
IQRBW	0.28	0.29	0.29	0.30	0.31	0.30	0.31	0.30	0.15	49.76	0.0–1.6	0.91	1.73
VBW	0.36	0.42	0.28	0.20	0.97	0.88	0.98	0.58	0.56	96.71	0.0–2.4	0.46	−1.17
SDBW	0.18	0.19	0.21	0.23	0.12	0.11	0.10	0.16	0.12	76.15	0.0–0.8	0.53	−0.24
CVBW	13.06	12.53	14.65	15.76	7.23	7.45	6.31	11.03	8.18	74.17	0.1–55.6	0.76	−0.12

TNB, total number born; NBA, number born alive; LBW, litter birth weight; MBW, mean birth weight; IBW, individual birth weight; range, maximum minimum birth weight; IQRBW, interquartile range of birth weight; VBW, variance of birth weight; SDBW, standard deviation of birth weight; CVBW, coefficient of variance of birth weight; 2 crossbred, pigs produced from a two-way cross between two purebred parental lines; Synthetic, pigs developed by interbreeding multiple breeds over generations to combine desirable traits from the parent breeds while maintaining genetic stability.

### 3.2 Appropriate fixed effects and heritability estimates

The models used for litter size, piglet birth weight, and uniformity of piglet birth weight traits are described in Table 2. For traits such as TNB and NBA, the significant fixed effects were the breed of piglets, body condition score of sows, breed of sows, parity of sows, month-year of farrowing, and sow feeding program. The random effects included direct additive, mate-sire, and permanent environmental effects. The analysis of piglet birth weight traits (LBW, MBW, and IBW) and uniformity traits (Range, IQRBW, VBW, SDBW, and CVBW) showed that fixed effects such as the breed of piglets, body condition score of sows, breed and parity of sows, month-year of farrowing, and sow feeding program made significant contributions. However, the fixed effect of the sex ratio between male and female piglets was significant only for the IBW trait. Random effects, including direct additive, maternal, and permanent environmental effects, also played an important role.

Table 4 shows the estimated heritability of litter size piglet birth weight, and uniformity of piglet birth weight traits. TNB and NBA traits had total variances of 7.345 and 6.191, respectively. Mate-sire genetic variance contributed significantly to these traits, with estimates of 0.422 for TNB and 0.436 for NBA. The residual variance was the largest component for both characteristics. Direct additive heritability was low for TNB (0.035 ± 0.01) and NBA (0.038 ± 0.01), while proportion of phenotypic variance explained by the mate-sire effect was higher, at 0.230 ± 0.05 for TNB and 0.243 ± 0.06 for NBA. LBW had the highest total variance (3.866) among the piglet birth weight traits, with direct additive genetic variance contributing 0.768 and maternal genetic variance contributing 0.650. The direct additive heritability was estimated at 0.199 ± 0.03, while maternal heritability was 0.168 ± 0.02. MBW showed the lowest variances compared to LBW and IBW, with direct additive heritability estimated at 0.183 ± 0.02 and maternal heritability at 0.030 ± 0.01. For individual birth weight (IBW), the direct additive heritability was 0.119 ± 0.02, and maternal heritability was 0.072 ± 0.01. Uniformity traits, including range, IQRBW, VBW, SDBW, and CVBW, had low total variances, ranging from 0.517 to 0.521. Maternal genetic variance and permanent environmental variance contributed minimally to these traits, suggesting a smaller genetic influence compared to environmental factors. The direct additive heritability estimates for these traits ranged from 0.069 to 0.075, while maternal heritability ranged from 0.046 to 0.050.

**Table 4:** Estimated variance components and heritability of litter size, piglet birth weight, and uniformity of piglet birth weight traits.

Traits	Variance components and estimated heritability								
	Vd	*Vs*	Vm	Vpe	Ve	Vt	h^2^d (±SE)	h^2^s (±SE)	h^2^m (±SE)
Litter size traits									
TNB	0.255	0.422	-	1.142	5.526	7.345	0.035 ± 0.01	0.230 ± 0.05	-
NBA	0.276	0.436	-	0.975	5.504	6.191	0.038 ± 0.01	0.243 ± 0.06	-
Piglet birth weight traits									
LBW	0.768	-	0.650	0.992	1.456	3.866	0.199 ± 0.03	-	0.168 ± 0.02
MBW	0.010	-	0.002	0.022	0.021	0.055	0.183 ± 0.02	-	0.030 ± 0.01
IBW	0.065	-	0.039	-	0.440	0.544	0.119 ± 0.02	-	0.072 ± 0.01
Uniformity of piglet birth weight traits									
Range	0.037	-	0.024	0.013	0.443	0.517	0.072 ± 0.02	-	0.046 ± 0.01
IQRBW	0.037	-	0.026	0.013	0.444	0.520	0.071 ± 0.02	-	0.050 ± 0.01
VBW	0.036	-	0.026	0.013	0.445	0.520	0.069 ± 0.02	-	0.050 ± 0.01
SDBW	0.038	-	0.025	0.013	0.444	0.520	0.073 ± 0.02	-	0.048 ± 0.01
CVBW	0.039	-	0.025	0.013	0.444	0.521	0.075 ± 0.02	-	0.048 ± 0.01

TNB, total number born; NBA, number born alive; LBW, litter birth weight; MBW, mean birth weight; IBW, individual birth weight; Range, within-litter weight maximum-minimum of birth weight; IQRBW, within-litter-weight interquartile of birth weight; VBW, within-litter weight variance of birth weight; SDBW, within-litter weight standard deviation of birth weight; CVBW, within-litter weight coefficient of variation of birth weight; Vd, direct additive genetic variance; Vs, mate-sire genetic variance; Vm, maternal genetic variance; Vpe, permanent environmental variance; Ve, residual variance; Vt, total variance; h^2^d (±SE), direct additive heritability (±standard error); h^2^s (±SE), proportion of phenotypic variance explained by the mate-sire effect (±standard error); h^2^m (±SE), maternal heritability (±standard error).

### 3.3 Genetic and phenotypic correlation

The genetic and phenotypic correlations among litter size, piglet birth weight, and uniformity of piglet birth weight traits are presented in Table 5. High positive genetic (0.945) and phenotypic (0.922) correlations were observed between TNB and NBA, indicating that these traits are genetically and phenotypically similar. LBW exhibited moderate positive genetic correlations with NBA (0.417) and TNB (0.401), while the phenotypic correlations were slightly weaker, at 0.337 and 0.301, respectively. In terms of uniformity traits, CVBW showed strong positive phenotypic (0.802) and genetic (0.824) correlations with SDBW, emphasizing their close association. Both traits were negatively genetically correlated with VBW (−0.727 for CVBW and − 0.705 for SDBW). In addition, MBW and IBW exhibited moderate positive genetic correlations (0.375) but negative genetic correlations with TNB and NBA, ranging from −0.299 to −0.152 and − 0.325 to −0.244. All uniformity of piglet birth weight traits were positively correlated with LBW, with genetic correlations ranging from 0.222 to 0.332. Phenotypic correlations were lower than genetic correlations, and the results of most correlations for many traits were consistent with genetic correlations, except for the phenotypic correlations between litter size and uniformity of piglet body weight.

**Table 5:** Genetic (below diagonal) and phenotypic (above diagonal) correlations between litter size, piglet birth weight, and uniformity of piglet birth weight traits.

Traits	TNB	NBA	LBW	MBW	IBW	Range	IQRBW	VBW	SDBW	CVBW
TNB	-	0.922	0.301	−0.112	−0.245	0.279	0.144	−0.051	0.011	0.055
NBA	0.945	-	0.337	−0.092	−0.233	0.289	0.134	−0.076	0.024	0.067
LBW	0.401	0.417	-	0.345	−0.009	0.289	0.102	0.077	0.005	−0.046
MBW	−0.299	−0.152	−0.005	-	0.421	−0.010	−0.034	0.279	−0.049	−0.221
IBW	−0.325	−0.244	0.511	0.375	-	−0.015	−0.056	0.324	−0.067	−0.261
Range	0.289	0.265	0.225	0.226	0.331	-	0.533	−0.115	0.436	0.455
IQRBW	0.224	0.200	0.223	0.301	0.143	0.576	-	−0.121	0.422	0.415
VBW	0.221	0.199	0.222	0.189	0.097	−0.094	−0.051	-	−0.756	−0.739
SDBW	0.179	0.189	0.249	0.177	0.010	0.543	0.464	−0.705	-	0.802
CVBW	0.310	0.313	0.332	0.306	−0.061	0.503	0.450	−0.727	0.824	-

TNB, total number born; NBA, number born alive; LBW, litter birth weight; MBW, mean birth weight; IBW, individual birth weight; Range, within-litter weight maximum-minimum of birth weight; IQRBW, within-litter weight interquartile of birth weight; VBW, within-litter weight variance of birth weight; SDBW, within-litter weight standard deviation of birth weight; CVBW, within-litter weight coefficient of variation of birth weight.

### 3.4 Selection indices

**Figure 1:**
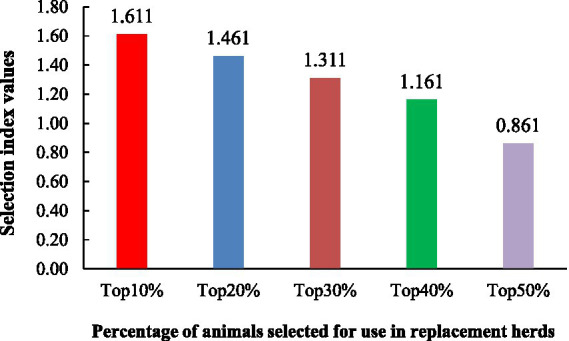
Top 10, 20, 30, 40, and 50% of the selection index values from the number born alive (NBA), litter birth weight (LBW), and the within-litter weight coefficient of variation of birth weight (CVBW) traits.

The selection index values for the number born alive (NBA), litter birth weight (LBW), and the within-litter coefficient of variation in birth weight (CVBW) were analyzed, highlighting the top 10, 20, 30, 40, and 50% of animals ranked by their index values (Figure 1). The selection of traits for constructing the selection index was based on heritability estimates, genetic correlations between traits, and economic importance, which aligned with the needs of pig farmers. NBA, LBW, and within-litter CVBW were the most prominent and suitable traits for inclusion in the selection index. Genetic parameters, including heritability estimates and genetic correlations between traits were used in this study. The marginal economic values (MEVs) for NBA, LBW, and CVBW were 0.36, 0.68, and − 0.76, respectively. The selection index (I) was defined as: $I=\left(0.2\times {\mathrm{EBV}}_{\mathrm{NBA}}\right)+\left(0.5\times {\mathrm{EBV}}_{\mathrm{LBW}}\right)+\left(0.3\times {\mathrm{EBV}}_{\mathrm{CVBW}}\right)$. Additionally, the percentage of animals selected for replacement herds indicated that the top 10% (1.611) had the highest selection index values compared to the top 20% (1.461), top 30% (1.311), top 40% (1.161), and top 50% (0.861).

## 4 Discussion

This study aimed to estimate genetic parameters and construct a selection index for litter size, piglet birth weight, and uniformity of birth weight in a commercial pig population in Thailand. The findings revealed that while heritability estimates for litter size traits were low, mate-sire genetic variance played a significant role in explaining variability. In contrast, piglet birth weight traits exhibited moderate heritability, with both direct additive and maternal genetic effects contributing to variation. Uniformity traits showed relatively low heritability, suggesting a stronger environmental influence. These results are consistent with previous studies that reported low genetic variability for litter size but moderate heritability for birth weight traits. The findings provide valuable insights into genetic improvement strategies for enhancing piglet survival and growth uniformity, which are crucial for commercial pig breeding programs. The following sections will discuss these findings in greater detail, considering the implications for genetic selection and management practices.

The observed differences in total number born (TNB) and number born alive (NBA) highlight the genetic potential of certain breed combinations, particularly Synthetic lines. The “Synthetic × 2 crossbred” combination exhibited the highest TNB (12.17) and NBA (11.37), suggesting that this crossbreeding strategy is effective in improving reproductive performance. These values align with previous studies in various pig breeds, such as Large White pigs in China (TNB = 10.46, NBA = 10.05) (24), Korean Duroc, Landrace, and Yorkshire pigs (TNB = 9.28–12.07, NBA = 8.28–11.04) (25), Large White sows in the Netherlands (TNB = 13.65, NBA = 12.56) (16), and in nine varieties of purebred and crossbred Iberian pig breeds (TNB = 7.59–8.84 and NBA = 7.21–8.55) (26). Moreover, the variability in TNB suggests that reproductive performance is influenced by both genetic and environmental factors (27). The slightly lower NBA (mean = 10.68) reflects a proportion of piglets not surviving the birthing process, underscoring the importance of improving survival rates alongside litter size (28). Piglet birth weight traits further emphasized the benefits of crossbreeding. The “Synthetic × 2 crossbred” combination displayed the highest mean litter birth weight (LBW) at 17.36 kg and a mean birth weight (MBW) of 1.55 kg, which are advantageous for piglet survival and growth. Low birth weights are associated with higher preweaning mortality and slower growth, emphasizing the need for strategies to improve uniformity in piglet litters (29, 30). Additionally, uniform litters have been shown to reduce preweaning mortality compared to heterogeneous ones (31, 32). However, within-litter uniformity, as reflected by the coefficient of variation in birth weight (CVBW), showed significant variability among breed combinations. The lower CVBW in Synthetic lines, particularly “Synthetic × 2 crossbred” (6.31%), indicates more uniform piglets, which can reduce competition for resources and improve survival rates (33).

Analysis of heritability estimates for litter size, piglet birth weight, and uniformity traits (Table 4) reveals a complex interplay of genetic and environmental factors that influences these economically important traits. Litter sizes, as represented by total number born (TNB) and number born alive (NBA), exhibited low heritability (0.035 ± 0.01 and 0.038 ± 0.01, respectively), indicative of a strong environmental influence (34). While additive genetic variance contributes, the substantial environmental variance highlights the significant potential for improvement through optimized management practices (35–37). The higher proportion of phenotypic variance explained by the mate-sire effect for NBA (0.243 ± 0.06) suggests a significant paternal influence on the number of piglets born alive, which is crucial for improving production efficiency in pig breeding (6). The identification of specific genetic markers and regions associated with NBA highlights the potential for paternal genetic contributions to influence this trait. This is supported by genome-wide association studies (GWAS) and other genetic analyses that have identified significant markers and candidate genes linked to NBA, suggesting a notable paternal genetic influence (38, 39).

In contrast, piglet birth weight traits (LBW, MBW, IBW) showed moderate to high heritability estimates (0.199 ± 0.03, 0.183 ± 0.02, and 0.119 ± 0.02, respectively), indicating the substantial potential for genetic improvement through selective breeding. The moderate maternal heritability for LBW and IBW highlights the importance of considering both sow genetics and management factors in optimizing birth weight. This finding, however, contrasts with Nguyen et al. (40), who reported lower maternal heritability than direct heritability for litter size and piglet weight. Conversely, Lee et al. (41) observed maternal heritability was twice as high as direct heritability. This discrepancy highlights the need for further research to elucidate the varying influence of maternal effects across different pig populations and production systems. However, optimizing birth weight requires a dual approach: genetic selection of superior sows combined with robust management practices, specifically focusing on sow nutrition. Optimal sow nutrition, particularly sufficient energy and essential micronutrients (e.g., vitamin B12, folate) during gestation, are crucial for fetal development and reducing the incidence of LBW (42, 43). Therefore, a comprehensive strategy to improve piglet birth weight must integrate both genetic selection of superior sows and meticulous management practices to optimize maternal condition and nutritional status throughout pregnancy. This dual approach is essential for enhancing neonatal outcomes and overall productivity in pig breeding programs.

Piglet birth weight uniformity traits (Range, IQRBW, VBW, SDBW, CVBW) demonstrated generally low heritability estimates (ranging from 0.069 to 0.075), confirming the predominant influence of environmental factors on within-litter variability (44). This aligns with previous research demonstrating the significant impact of sow nutrition and environmental conditions on piglet weight variation. Larger litter sizes are associated with increased birth weight heterogeneity and reduced average birth weight due to resource competition among piglets (45). Furthermore, sow nutritional status during gestation, particularly the availability of essential nutrients, plays a critical role in fetal development and reducing birth weight variation (46). Additional environmental factors, including uterine capacity and placental efficiency, also contribute to this variability (46). While management strategies such as split suckling and supplemental feeding can mitigate weaning weight variability (47), their impact on overall growth remains limited. Therefore, improving piglet birth weight uniformity necessitates a multi-faceted approach integrating improved sow nutrition and management practices to minimize environmental influences. One key aspect distinguishing this study from previous research is that it includes maternal genetic effects in evaluating piglet birth weight traits (LBW, MBW) and birth weight uniformity traits (Range, IQRBW, VBW, SDBW, CVBW). In contrast, earlier studies did not consider maternal genetic effects in their models. We hypothesized that incorporating maternal genetic effects into the genetic parameter estimation of piglet birth weight and uniformity would provide a more comprehensive and biologically meaningful assessment. Maternal factors, such as litter size, uterine crowding, and placental development, influenced milk production and postnatal care, which in turn affected piglet survival and birth weight uniformity (31, 40, 48, 49). These factors should have been accounted for in genetic evaluations. This study improved the selection accuracy for piglet viability and birth weight uniformity by including maternal genetic effects, ultimately contributing to enhanced productivity in swine breeding programs. Moreover, considering these effects enhanced model accuracy and provided more precise estimates of genetic parameters for these traits (50–52).

Our study revealed notable genetic and phenotypic correlations among various traits (Table 5). The strong positive genetic correlation between the total number born (TNB) and the number born alive (NBA) (*r* = 0.922) is expected, indicating that increasing TNB generally leads to a higher NBA. However, the phenotypic correlation (*r* = 0.945) is even stronger, suggesting that an increase in the total number of births typically results in a higher number of live births. This correlation is supported by studies focusing on the genetic architecture of these traits (51, 53). Meanwhile, the genetic correlations between litter size (TNB and NBA) and piglet birth weight traits (LBW, MBW, IBW) were predominantly negative. This means that larger litters are associated with lower average birth weights, as previous studies have shown that sows that produce high litter weights often produce piglets with lower IBWs (52, 54). These negative correlations indicate that selecting for larger litters may indirectly lead to smaller piglets. The genetic correlations between different birth weight traits (LBW, MBW, IBW) were positive and relatively high, suggesting that selection for one birth weight trait is likely to influence the others positively. This is supported by studies that have examined genetic parameters and correlations in various species, indicating a shared genetic basis for these traits (55). However, the genetic correlations between birth weight and birth weight uniformity traits (Range, IQRBW, VBW, SDBW, CVBW) show a more complex pattern. Some correlations are positive, suggesting that an increase in average birth weight might be associated with increased variability, while other correlations are negligible or negative. This indicates that increasing average birth weight might not necessarily lead to improved birth weight uniformity. Phenotypic correlations often show similar patterns to genetic correlations, although their magnitudes are often lower, highlighting the influence of environmental factors on these relationships.

Figure 1 illustrates the selection index values for different selection intensities (top 10–50%) based on a combination of the number born alive (NBA), litter birth weight (LBW), and within-litter weight coefficient of variation of birth weight (CVBW). Owing to the importance of these traits, the relative economic values of the NBA, LBW, and within-litter weight CVBW at birth were 0.2, 0.5, and 0.3, respectively. Although NBA has low heritability (0.038), the number of piglets born alive is crucial for productivity. Although nongenetic factors considerably influence this trait, more piglets can lead to higher profits. LBW has the highest relative economic value because a higher birth weight generally indicates healthier piglets and better survival rates, which directly affect profitability. LBW was more responsive to genetic selection, with moderate heritability (0.199). Finally, the CVBW is crucial for achieving uniformity within the litter, which affects management practices and market weight consistency. Despite its low heritability (0.075), reducing variation is economically beneficial, and leads to uniform growth and fewer management challenges. As expected, the selection index values decreased with increasing selection intensity, illustrating diminishing returns from less stringent selection (56). The top 10% (1.611) exhibited significantly higher index values than the top 50% (0.861), demonstrating the effectiveness of the index in identifying superior animals and the potential for genetic improvement through focused selection. This index effectively balances litter size, birth weight, and uniformity. Breeding programs must consider the trade-off between rapid genetic gain (achieved with stricter selection) and the preservation of genetic diversity (favored by broader selection). The choice of selection intensity will thus depend on the specific breeding goals and the balance between short-term gains and long-term population health.

The heritability estimates (Table 4), genetic correlations (Table 5), and the resulting selection index (Figure 1) illustrate the challenge of disentangling the effects of genetic and environmental factors on economically important piglet production traits. The low heritability of uniformity traits, despite their economic value, resulted in a lower weighting of CVBW within the selection index. This reflects the inherent challenge of achieving genetic improvement for uniformity, while still acknowledging its importance. Conversely, the moderate-to-high heritability of birth weight traits (LBW, MBW, IBW) and their strong positive genetic correlations justified their greater weighting in the index, highlighting the potential for effective genetic gains through selective breeding. The negative genetic correlations between litter size and birth weight (Table 5) highlighted the inherent trade-off between these traits, a challenge directly addressed in the design of the selection index (Figure 1). This integrated approach, which considers litter size, birth weight, and uniformity, aims to increase overall farm efficiency. Therefore, breeders should prioritize birth weight in selection programs while simultaneously implementing robust management practices to address the significant environmental influences on litter size and uniformity. Future research should focus on identifying genetic markers for uniformity and elucidating the complex interplay of genetic and environmental factors affecting these key production traits to optimize both selection indices and management practices.

While this study highlights the effectiveness of a multiple-trait animal model and selection index, some limitations should be addressed to improve its application. First, traits with low heritability, such as NBA and CVBW, are significantly influenced by non-genetic factors. Future studies should integrate non-additive genetic effects, such as epistasis, which have been shown to improve genomic prediction accuracy for NBA by up to 12% (57). Second, environmental and management factors can vary across pig farms, and accurately accounting for these differences is essential to ensure precise genetic parameter estimates. Finally, the economic weights in the index were determined based on current genetic parameters and economic conditions, which may vary across production systems. Future studies could explore dynamic weighting systems to adapt to changing market demands and production scenarios.

In conclusion, to enhance litter size and uniformity in this pig population, it is essential to prioritize NBA, LBW, and CVBW in genetic improvement programs. Estimating genetic parameters allows breeders to make informed decisions when developing selection indices, ultimately leading to larger, more uniform litters. This is particularly beneficial for commercial pig farming where productivity directly affects profitability. Research indicates that uniform birth weight is linked to improved postnatal growth performance and reduced piglet mortality because lighter piglets are more vulnerable. Focusing on litter size and birth weight uniformity in selection indices aligns with sustainable farming practices by optimizing resource use and promoting more efficient production systems.

## Data Availability

The original contributions presented in the study are included in the article/supplementary material, further inquiries can be directed to the corresponding author.
